# Systematic analysis of plasmids of the *Serratia marcescens* complex using 142 closed genomes

**DOI:** 10.1099/mgen.0.001135

**Published:** 2023-11-15

**Authors:** Debora Satie Nagano, Itsuki Taniguchi, Tomoyuki Ono, Keiji Nakamura, Yasuhiro Gotoh, Tetsuya Hayashi

**Affiliations:** ^1^​ Department of Bacteriology, Graduate School of Medical Sciences, Kyushu University, Higashi-ku, Fukuoka, 812-8582, Japan; ^2^​ Department of Cardiovascular Surgery, Graduate School of Medical Sciences, Kyushu University, Higashi-ku, Fukuoka, 812-8582, Japan

**Keywords:** closed genome, GC content, host range of plasmid, plasmid, *Serratia marcescens* complex

## Abstract

Plasmids play important roles in bacterial genome diversification. In the *Serratia marcescens* complex (SMC), a notable contribution of plasmids to genome diversification was also suggested by our recent analysis of >600 draft genomes. As accurate analyses of plasmids in draft genomes are difficult, in this study we analysed 142 closed genomes covering the entire complex, 67 of which were obtained in this study, and identified 132 plasmids (1.9–244.4 kb in length) in 77 strains. While the average numbers of plasmids in clinical and non-clinical strains showed no significant difference, strains belonging to clade 2 (one of the two hospital-adapted lineages) contained more plasmids than the others. Pangenome analysis revealed that of the 28 954 genes identified, 12.8 % were plasmid-specific, and 1.4 % were present in plasmids or chromosomes depending on the strain. In the latter group, while transposon-related genes were most prevalent (31.4 % of the function-predicted genes), genes related to antimicrobial resistance and heavy metal resistance accounted for a notable proportion (22.7 %). Mash distance-based clustering separated the 132 plasmids into 23 clusters and 50 singletons. Most clusters/singletons showed notably different GC contents compared to those of host chromosomes, suggesting their recent or relatively recent appearance in the SMC. Among the 23 clusters, 17 were found in only clinical or only non-clinical strains, suggesting the possible preference of their distribution on the environmental niches of host strains. Regarding the host strain phylogeny, 16 clusters were distributed in two or more clades, suggesting their interclade transmission. Moreover, for many plasmids, highly homologous plasmids were found in other species, indicating the broadness of their potential host ranges, beyond the genus, family, order, class or even phylum level. Importantly, highly homologous plasmids were most frequently found in *Klebsiella pneumoniae* and other species in the family *Enterobacteriaceae *, suggesting that this family, particularly *K. pneumoniae *, is the main source for plasmid exchanges with the SMC. These results highlight the power of closed genome-based analysis in the investigation of plasmids and provide important insights into the nature of plasmids distributed in the SMC.

## Impact Statement

Plasmids are important players in bacterial genome diversification. While growing numbers of bacterial draft genomes are rapidly accumulating, accurate analyses of plasmids in draft genomes are not easy. Here, we describe the results of a systematic plasmid analysis of the *Serratia marcescens* complex (SMC) using 142 closed genomes. We identified 132 plasmids in 77 strains, which were separated into 23 clusters and 50 singletons based on their pairwise Mash distances. A systematic analysis of these plasmids provided multiple lines of findings important for better understanding the nature of plasmids in the SMC. They included the notable difference in GC content between most plasmids and their host chromosomes, which suggests their recent appearance in the SMC, the intra-complex transmission of the SMC plasmids, and their broad potential host ranges beyond the genus, family, order, class or even phylum level. Moreover, members of the family *Enterobacteriaceae,* particularly *Klebsiella pneumoniae *, were suggested as the main sources for plasmid exchanges with the SMC. These results show the power and importance of closed genome-based analysis in better understanding the nature of plasmids in bacterial populations.

## Data Summary

The raw read sequences and closed genome sequences obtained in this study have been deposited in GenBank/EMBL/DDBJ under the BioProject accession number PRJDB10568 (see also Table S1, available in the online version of this article, for the accession numbers of each strain), and the Sequence Read Archive (SRA) accession numbers DRR493224 to DRR493297. All supporting data have been provided within the article or through supplementary data files.

## Introduction

*Serratia marcescens* (Sma) is a Gram-negative, rod-shaped bacterium ubiquitous in the environment but has attracted attention as an opportunistic pathogen in the last few decades. It causes a wide range of nosocomial infections, such as pneumonia, bacteraemia, meningitis and endocarditis, including outbreaks, in compromised patients, particularly in neonatal and other intensive care units [1–7]. Sma strains have also been isolated from hospital equipment (e.g. ventilators) and the environment (e.g. sinks) and spread by healthcare professionals, as recently described in hospitalized COVID-19 patients in a Portuguese hospital [8]. Moreover, in addition to the natural resistance of Sma to multiple antimicrobial classes, such as penicillins and first- and second-generation cephalosporins, their acquisition of mobile genetic elements (MGEs), primarily plasmids, carrying multiple antimicrobial resistance (AMR) genes has raised further public health concerns as their acquisition limits treatment options, making Sma strains potentially more harmful to compromised patients [6, 7].

Sma was formerly included in the family *Enterobacteriaceae* but is now classified as a member of the family *Yersiniaceae* [9]. Recently, we performed a whole genome sequence (WGS)-based analysis of a global set of Sma, including *S. marcescens* subsp. *sakuensis *, and its close relatives, *Serratia nematodiphila* (Sne), *Serratia ureilytica* (Sur), and *Serratia surfactantfaciens* (Ssu), collectively named the Sma complex (SMC). This analysis identified 14 clades with an average nucleotide identity (ANI) threshold of 97 %, four of which corresponded to Sma *sensu stricto* (clade 11), Sne (clade 10), Sur (clade 12) and Ssu (clade 13) [6]. Importantly, many strains isolated from patients and hospital environments and identified as Sma in laboratories formed two clades (clades 1 and 2) distinct from the other clades, and the strains belonging to these two clades contained more AMR genes. Moreover, clade 2 strains have larger genomes with lower GC contents than the strains in the other clades, and it was suggested that this feature was due to the acquisition of more plasmids and integrative elements because more plasmid replicons and integrase genes were detected in clade 2 strains [6]. In several other clades, there were also negative correlations between genome size and GC content and positive correlations between genome size and the number of plasmid replicons and/or integrase genes. Similar findings were obtained by WGS analysis of the entire genus *Serratia* [10]. However, the draft genome sequences of MGEs, particularly those of large plasmids, are often fragmented [11, 12]. Thus, although several plasmid analysis tools based on replication systems and/or mobilization systems are now available, it is not easy to identify their sequences [13, 14]. This was also the case for the SMC strains, for which intensive plasmid analyses similar to those for the pathogens belonging to *Enterobacteriaceae *, such as *Escherichia coli* and *Klebsiella pneumoniae* [15, 16], have not yet been conducted. Therefore, accurate repertoires and natures of the plasmids in the SMC remain elusive.

In this study, to overcome the limitations of draft genome sequences in plasmid analysis, we obtained 67 SMC closed genome sequences and collected 75 SMC closed genomes from the National Center for Biotechnology Information (NCBI) database. In these closed genomes, we identified 132 plasmids in 77 genomes, which were separated into 23 clusters and 50 singletons based on their pairwise Mash distances. We performed systematic analyses of these plasmids, including of their sizes and GC contents, analysed relationships with the phylogeny of host strains, and conducted a database search for highly homologous plasmids outside the SMC. These analyses provided multiple lines of important findings on the nature of SMC plasmids, such as their potentially broad host ranges beyond family, order, class or even phylum level.

## Methods

### Strain set, long-read sequencing and hybrid assembly to closure

Of the 142 closed genomes analysed in this study, 75 were obtained from the NCBI database (accessed 10 December 2021). The remaining 67 genomes were closed in this study. To obtain these 67 closed genomes, we initially selected 77 strains from a collection of 227 SMC strains that were previously draft-sequenced [6] based on the clades of strains, the results of plasmid detection by local PlasmidFinder v2.0.1 [17] and MOBScan server (https://castillo.dicom.unican.es/mobscan/) [18], and an AMR gene search by SRST2 v0.2.0 [11] with the ARG-ANNOT_v3 database (https://www.mediterranee-infection.com/wp-content/uploads/2019/03/arg-annot-nt-v3-march2017.txt) to capture the diversity of plasmids and host strains as much as possible. Genomic DNA of the 77 strains was purified with the QIAGEN Genomic Tip 100 G^−1^ kit (Qiagen) according to the manufacturer’s instructions, except for the incubation temperature (55 °C). When we failed to obtain high-molecular-weight (HMW) DNA, an additional step of 20 min of incubation at 65 °C was included prior to the lysis step to prevent autodegradation [19]. As HMW DNA was not obtained from 10 strains even with this additional incubation, these strains were excluded from the subsequent analysis. The successfully purified genomic DNA of 67 strains was subjected to long-read sequencing by the Oxford Nanopore Technologies (ONT) sequencing technology. Libraries were prepared using the Rapid Barcoding Kit (ONT) and sequenced using MinION R9.4.1 flow cells (ONT). Sequence reads were base-called using the Albacore v2.3.1 and quality-filtered and adapter-trimmed (score: >10, read length: ≥2000 bp, initial 100 bp of reads trimmed) using the Nanofilt v2.7.1 [20]. When the long-read coverage was >100×, reads were half sampled using the SeqKit toolkit v2.3.0 [21]. Then, by using Unicycler v0.4.8 [22], closed genomes were obtained by hybrid assembly using the long-read sequences obtained in this study and the Illumina short reads previously obtained [6], except for seven strains. As the amounts and qualities of the Illumina short reads from these seven strains appeared insufficient to obtain closed genomes, these strains were sequenced again on the Illumina MiSeq platform using the libraries prepared with the NEBNext Ultra II FS DNA Library Prep Kit (New England Biolabs), and the obtained Illumina short reads were used for hybrid assembly to obtain their closed genomes. The position of nucleotide 1 of the chromosome sequences was adjusted to that of strain Db11 [1], and gene annotation was conducted using the DNA Data Bank of Japan (DDBJ) Fast Annotation and Submission Tool (DFAST) v1.2.0 [23].

### Replicon and MOB typing of plasmids

We identified all extrachromosomal sequences in the 142 closed genomes, and then their replicon and MOB typing was performed via Mob-Typer v3.0.3 from the Mob-suite package [24] for both typings, local PlasmidFinder v2.0.1 [17] for replicon typing and MOBScan server (https://castillo.dicom.unican.es/mobscan/) [18] for MOB typing. MOB subfamilies were determined based on the previously described conserved residues and motifs of relaxases [14, 25], the results of BLASTp sequence similarity analysis with prototype relaxases, and neighbour-joining (NJ) phylogenetic trees reconstructed for each subfamily using the alignments generated by ClustalW [26] in MEGAX [27]. Truncated relaxases were excluded from the analysis. A diagram showing the correlation of replicon and MOB types was generated using the package networkD3 in R v4.2.3 [28].

### Phylogenetic analyses

The clades of 52 closed genomes obtained from the NCBI database were unknown because they were not included in our previous analysis [6]. Therefore, their clades were determined by a core gene-based phylogenetic analysis of these 52 closed genomes, three draft genomes of type strains, the 775 previously analysed genomes [6] and *Serratia ficaria* NCTC 12148 (used as an outgroup). We first determined the core genes (*n*=2429) of these 831 genomes using Roary v.3.12 [29] with an 80 % BLASTp identity cutoff and generated a core-gene sequence alignment using mafft, as previously described [6]. Based on the 193 503 SNPs identified in this alignment, we reconstructed a maximum-likelihood (ML) tree using RAxML v8.2.10 [30] with the GTRGAMMA model of nucleotide substitution and 100 bootstrap replicates (Fig. S1). Based on this result, we determined the clades of the 52 closed genomes newly obtained from NCBI and the three type strains. Afterwards, we repeated the phylogenetic analysis using only 146 closed genomes, which consisted of 67 genomes closed in this study and 79 closed genomes from the NCBI database. As four pairs of genomes in the NCBI genome set were identical in core genome sequence and did not carry plasmids, only one of each pair was included in further analyses. Thus, our final dataset included 142 closed genomes, from which we obtained 535 875 SNPs, in the sequence alignment of 3189 core genes, and the ML tree of these 142 genomes was reconstructed as described above.

An additional ML tree including only 77 strains that carried plasmids was reconstructed based on the 565 456 SNPs in 3311 core genes which were identified using the same tools and parameters as described above. This tree was used to visualize the correlation of the results of plasmid clustering with the phylogeny of host strains using the cophylo command of the phytools package in R v4.2.3 [28].

### Pangenome analysis

The pangenome of the 142 closed genomes was determined by Roary v3.12 [29] with a 95 % BLASTp identity for gene clustering to construct a presence/absence matrix. Based on the presence/absence matrix of the genes in the pangenome, genes were classified into three groups: (i) those specifically encoded by chromosomes, (ii) those specifically encoded by plasmids, and (iii) those encoded by chromosomes or plasmids depending on the strain. Annotations of the genes in the second and third groups were manually curated based on the results of the BLASTp homology search of the NCBI database.

### Search for AMR and heavy metal resistance genes

AMR and heavy metal resistance (HMR) genes were identified with AMRFinderPlus v3.10.30, database v2022-05-26, with the --plus option [31]. The AMR genes and HMR genes identified were further manually checked for variant and operon composition, respectively.

### Analysis of linear plasmids

In two genomes that we tried to close in this study, a contig sequence that appeared to represent a linear plasmid was generated in addition to a circular closed chromosome. To exclude the possibility that they were derived from gap-containing circular plasmid sequences, PCR analyses were performed using two sets of primers designed to fill the potential gaps between ends in each contig (Fig. S3). Non-digested genomic DNA of the two strains was also analysed by PFGE to detect linear extra-chromosomal DNA molecules using a 1 % agarose gel prepared with Certified Megabase agarose (Bio-Rad) and the CHEF Mapper XA system (Bio-Rad) at 14 °C and 6 V cm^–1^ for 20 h. Pulse times were ramped with 0.5 s at the beginning and 30 s at the end [19, 32, 33]. The gel was stained with SYBR Safe DNA Gel Stain (Thermo Fisher Scientific), and images were obtained using the Gel Doc XR Imaging Systems (Bio-Rad). The genome maps of the two linear plasmids were generated with GenomeMatcher v3.04 [34] and their nucleotide sequence identity was determined by BLASTn.

### Mash distance-based clustering of plasmids

All-against-all Mash distances of the 132 plasmids were calculated using Mash v2.0 with default k-mer and sketch sizes [35]. Based on the distance matrix constructed, the plasmids were clustered by the complete linkage method with the hclust command and a cut-off of 0.06 [24]. The ggtree package in R v4.2.3 [28] was used for dendrogram construction and dataset visualization. Network diagrams of the 23 plasmid clusters were prepared and visualized with Cytoscape v3.10.0 [36] using Prefuse Force Directed layout weighted by pairwise Mash distances with default parameters.

### Database search for plasmids highly homologous to the SMC plasmids

We initially searched the NCBI database for sequences highly homologous to the plasmids identified in this study using BLASTn with an identity threshold of ≥90 %. After excluding the sequences labelled ‘* Serratia marcescens* (taxid:615)’, ‘* Serratia ureilytica* (taxid:300181)’, ‘* Serratia nematodiphila* (taxid:458197)’ and ‘*Serratia surfactantfaciens* (taxid:2741499)’, and those not indicated as plasmids, Mash distances of the remaining sequences from corresponding SMC plasmid sequences were calculated as described above, and those showing Mash distances <0.06 were selected as highly homologous plasmids.

### Statistical analyses

Statistical analyses were performed in R v4.2.3 [28]. For the comparison of the average numbers of plasmids carried by strains belonging to different clades, one-way ANOVA followed by a Tukey–Kramer multiple comparison test was used. For the comparison of the average numbers of plasmids carried by strains from clinical/hospital environments and non-clinical sources, the Mann–Whitney U test was used. Simple linear regression analysis was used to analyse the relationship between plasmid GC content and plasmid size.

## Results and discussion

### Closed genome set

The closed genome set analysed in this study included 75 genomes obtained from the NCBI database and 67 genomes closed in this study. The clades of the 52 genomes from the NCBI database, which were not included in our previous study [6], were determined by core gene-based phylogenetic analyses with the SMC genomes used in our previous study. The 67 genomes closed in this study were selected to cover the entire complex and the variation in AMR gene repertoires in the complex as much as possible. The clade distribution of the 142 genomes and their isolation sources are summarized in Table 1 (see also Table S1 and Fig. S2 for details of each genome). Although some of the clades were poorly represented in this genome set, the number of strains belonging to these clades was small in the whole set (see Fig. S1 for the phylogenetic positions and clades of the 142 strains in the whole set of SMC strains). They were isolated from five geographical regions: Japan (*n*=58), North America (*n*=37), Europe (*n*=23), Asia excluding Japan or Oceania (*n*=23), and South America (*n*=1). The average size and GC content of the chromosomes were 5.23 Mb (4.93–5.64 Mb) and 59.58 % (58.92–60.16 %), respectively (Fig. S2). As expected, the total sizes of the 67 genomes closed in this study were larger than those of their draft sequences with an average difference of 38.9 kb (0.7–108.0 kb) (Table S2).

**Table 1.** Clades and isolation sources of the strains analysed

Clade*	No. of strains	Clinical/non-clinical
1	33	33/0
2	11	10†/1
3	10	7/3
4	6	6/0
5	1	0/1
6	5	0/5
7	3	0/3
8	2	0/2
9	11	6/5
10 (Sne)	13	4/9
11 (Sma *s.s*.)	14	5/9
12 (Sur)	29	12/17
13 (Ssu)	4	0/4

*Sne; *S. nematodiphila,* Sma *s.s.; S. marcescens sensu stricto*, Sur; *S. ureilytica*, Ssu; *S. surfactantfaciens*.

†Including two hospital environmental strains.

### Plasmids in the 142 closed genomes

**Fig. 1.**
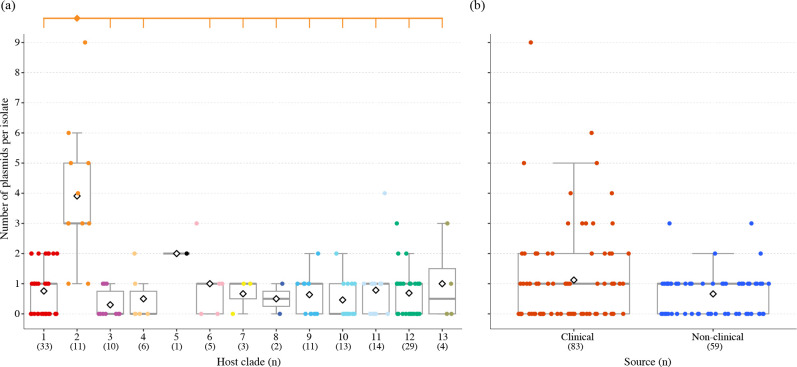
The numbers of plasmids identified in the 142 closed genomes. (**a**) Comparison of the numbers of plasmids carried by SMC strains belonging to 13 clades. Open diamonds in the boxplot indicate mean values. A closed diamond indicates the clade which showed a significant difference (*P*<0.01) from the clades indicated by short vertical lines. There was no significant difference between the 12 clades other than clade 2. (**b**) Comparison of the numbers of plasmids carried by the clinical/hospital environment and non-clinical strains. Open diamonds in the boxplot indicate mean values. A statistically significant difference was not detected between the two groups.

**Fig. 2.**
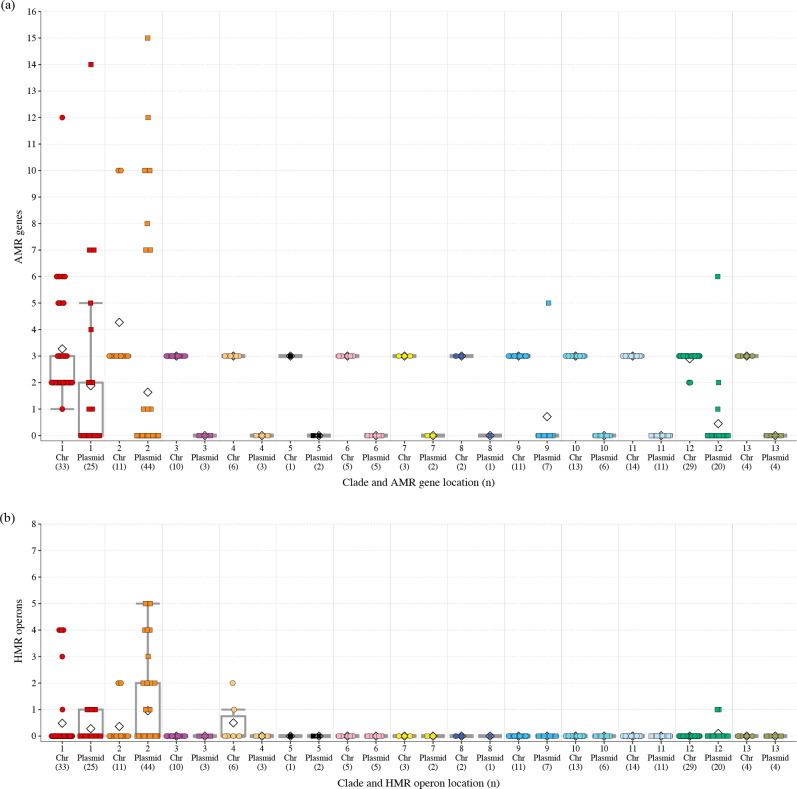
AMR genes and HMR operons identified in the 142 closed genomes. The cumulative numbers of AMR genes (**a**) and HMR operons (**b**) in the chromosomes (circles) and plasmids (squares) of each strain are shown.

We identified 132 plasmids in the genomes of 77 strains, ranging from 1.4 to 244.4 kb in size with 71.9 kb on average (Table S1 and S3, and Fig. S2). Consistent with our previous result [[6]], the average number of plasmids found in the strains in clade 2, a hospital-adapted clade, was significantly higher than that in other clades (Fig. 1a). Most clade 2 strains contained three or more plasmids, with one containing as many as nine plasmids, whereas two clade 2 strains contained only one plasmid (Fig. S2), of which one was from the natural environment (water) and contained only a small plasmid 5.3 kb in size (Table S3). In contrast, the strains in clade 1, another hospital-adapted clade, did not contain more plasmids than the other clades, which was also consistent with our previous results [6]. Consequently, the average numbers of plasmids between clinical (*n*=83; including two strains isolated from the hospital environment) and non-clinical strains (*n*=59) were not significantly different (*P*=0.316; Fig. 1b). However, when the total numbers of AMR genes and HMR operons encoded in each clade were analysed, notable differences were observed between the two hospital-adapted clades and the other clades (Fig. 2). Although we identified 49 AMR genes and six HMR operons in the whole dataset (Table S1), the same pair of AMR genes [*aac(6’)-Ic* and *bla*_SRT_)] was encoded in the chromosomes, and no genes/operons were encoded by the plasmids in most strains belonging to clades other than clades 1 and 2. In contrast, the numbers of these AMR genes and HMR operons in clades 1 and 2 were highly variable in both chromosomes and plasmids, indicating the acquisition of these genes/operons by these clades via horizontal gene transfer (HGT) mediated by both plasmids and some integrative elements.

### Linear plasmids

The 132 plasmids included two similar linear plasmids 43.1 and 36.5 kb in size, showing >95 % ANI values (Fig. S3). The linearity of their genomes was verified by PCR analysis with primers targeting each end of the genomes (see Fig. S3a for their positions), which yielded no amplicons (Fig. S3b). In addition, by PFGE analysis of undigested whole-genomic DNA of the two strains containing these linear plasmids, an extrachromosomal DNA band with the expected size was detected in both strains (Fig. S3c). Although we were unable to determine the exact terminal sequences of their genomes, a terminal inverted sequence of at least 490 bp was identified. Moreover, the two plasmids showed >90 % nucleotide sequence identity with the linear plasmids previously identified in *Klebsiella* and other *Enterobacteriaceae* [37].

### Replicon and MOB types of plasmids

**Fig. 3.**
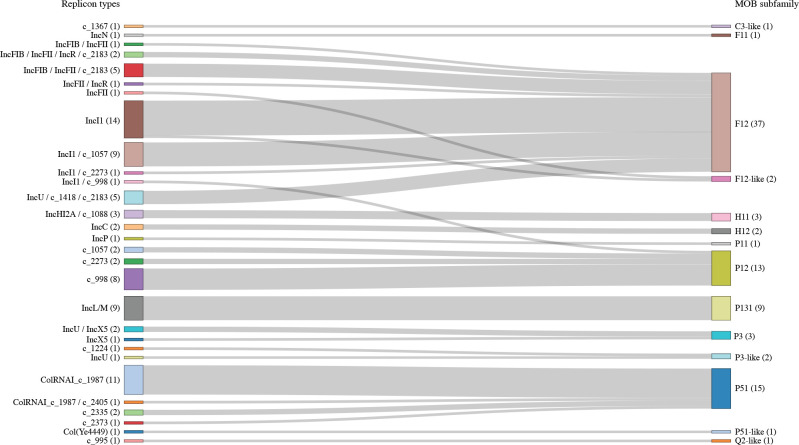
Combinations of plasmid replicon types and MOB subfamilies. A Sankey diagram presenting the combinations of replicon types (left) and MOB subfamilies (right) identified in the 90 SMC plasmids, in which both replicon type and MOB subfamily were defined, is shown. The numbers of each replicon type and MOB subfamilies are indicated in parentheses. Replicon types indicated by c_XXXX are those defined as rep_cluster_XXXX by MOB-Typer.

To determine the replicon and MOB types of the 132 plasmids, while we initially used three typing tools, PlasmidFinder, MOBScan and MOB-Typer, the largest number of plasmids were typed by MOB-Typer (Table 2; see Table S3 for the initial outputs of three tools). Therefore, we used the results predicted by this tool in this study. The replicon types of 108 plasmids (including uncharacterized types labelled ‘rep_cluster’) were determined by MOB-Typer (see Table S3 for details). Of the typed plasmids, 33 contained multiple replicons. Among the 24 untypeable plasmids, 13 were small plasmids (1.9–5.1 kb), but the remaining 11 ranged from 13.9 to 139.9 kb in size. They included the two linear plasmids, which probably use a 5′ terminus-binding protein as a primer for replication [37, 38]. The MOB types of 112 plasmids were determined by MOB-Typer. All known MOB families (MOB_C_, MOB_F_, MOB_H_, MOB_P_, MOB_Q_ and MOB_V_) were detected in the 112 plasmids, and the MOB subfamilies were further determined for the 105 plasmids that encoded intact relaxases based on the sequence homologies of their protein sequences (Table S3). Of the 27 untypeable plasmids, 13 were small plasmids (1.9–6.3 kb). The remaining 14 plasmids, which included the two linear plasmids, ranged from 11.1 to 101.5 kb in size. The combinations of MOB types and replicon types were generally consistent with known combinations (MOB_F_-IncF/IncN; MOB_H_-IncH/IncC; MOB_P_-IncP/Col/IncX/IncU) [14, 39], but most plasmids encoding the IncI1 replicon also contained the MOB_F12_ subfamily relaxase genes and only one IncI1 plasmid contained the MOB_P12_ subfamily relaxase gene (Fig. 3).

**Table 2.** Replicon and MOB typing of 132 SMC plasmids by three typing tools

Typing tool	Replicon		MOB	
	Detected	Not detected	Detected	Not detected
MOB-Typer	108	24	112†	20
PlasmidFinder	69*	63	–	–
MOBScan	–	–	107‡	25

*All replicons detected by PlasmidFinder were also detected by MOB-Typer.

†Of these 112 plasmids, 105 contained apparently intact relaxases, the MOB subfamilies of which were determined as described in the Methods and used for further analyses.

‡Of these 107 plasmids, 103 contained apparently intact relaxases. Their MOB subfamilies were also determined as described in the Methods and used for further analyses. Note that the 103 plasmids were included in the 105 plasmids typed by MOB-typer.

### Genes specific to plasmids and those encoded by chromosomes or plasmids

**Fig. 4.**
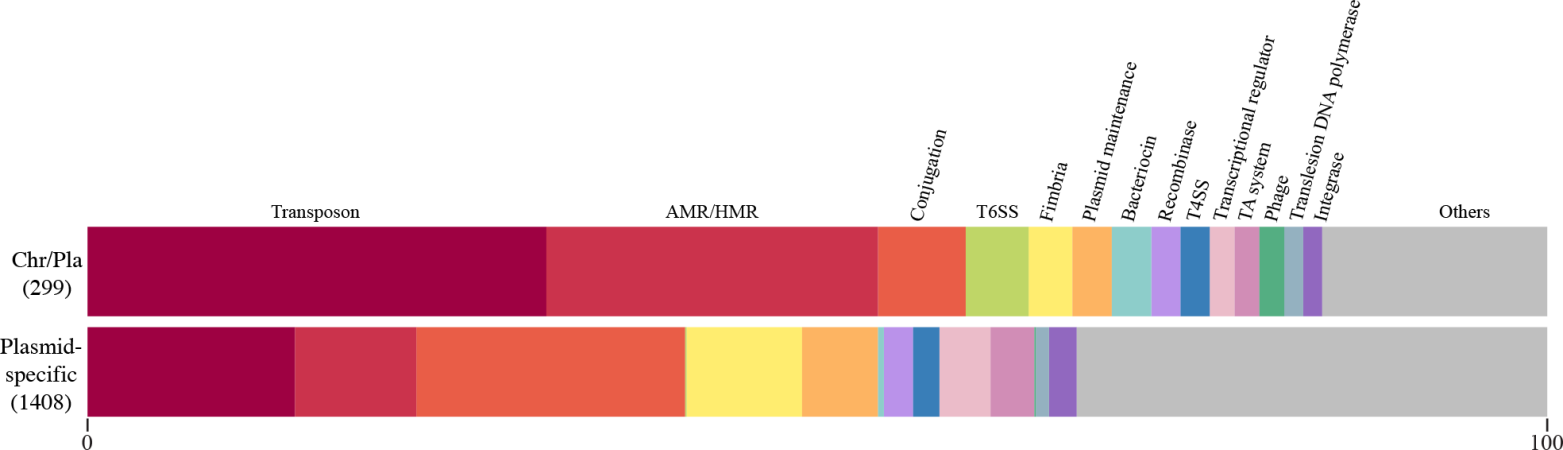
Functions of the genes encoded by chromosomes or plasmids (Chr/Pla genes). The proportions of 14 functional gene groups containing four or more members in the 299 function-predicted Chr/Pla genes are shown. For comparison, the proportions of these 14 gene groups in the 1408 function-predicted plasmid-specific genes are also shown. Note that while ‘Others’ in the Chr/Pla genes included gene groups containing fewer than four members, ‘Others’ in the plasmid-specific genes included not only those containing fewer than four members but also those including four or more members (10 or fewer members in all but one group). Hypothetical genes were not included in this analysis.

To identify the genes specifically encoded by plasmids and those encoded by chromosomes or plasmids depending on the strain, we performed a pangenome analysis of the 142 genomes by Roary v3.12 [29] with a 95 % sequence identity threshold. Among the 28 954 genes identified, 24 858 (85.8 %) were specifically present in chromosomes, 3693 (12.8 %) were specific to plasmids, and the remaining 403 (1.4 %) were present in either chromosomes or plasmids (referred to as ‘Chr/Pla genes’; listed in Table S4). While a large proportion (*n*=2285, 61.9 %) of the plasmid-specific genes encoded hypothetical proteins of unknown function, genes for conjugation (*n*=259) were most predominant among the 1408 function-predicted genes, followed by genes related to transposon (Tn) (*n*=200), AMR/HMR (*n*=117) and fimbria formation (*n*=111) (Fig. 4). Among the 299 function-predicted Chr/Pla genes, while Tn-related genes were most predominant (*n*=94), AMR/HMR genes (*n*=68) were also enriched (Fig. 4), indicating that a considerable number of AMR/HMR genes move between chromosomes and plasmids. Of note, genomic locations (chromosome or plasmid) of many genes were variable even between closely related strains (see Fig. S4 for the phylogeny of host strains and the genomic locations of the 299 genes in each strain). Consistent with this, the Chr/Pla genes also included notable numbers of conjugation-related genes (*n*=18), type 4 secretion system (T4SS)-related genes (*n*=5), which can also be involved in conjugation [40], and genes for plasmid maintenance (*n*=8), recombinases (*n*=6) and integrases (*n*=4) (see Table S4 and Fig S4 for details), suggesting that these AMR/HMR genes may be encoded by integrative conjugative elements (ICEs) and other integrative elements such as integrative plasmids. This result may partly explain why more AMR genes and HMR operons accumulated in clade 1 strains, because some of the clade 1 strains contained many AMR genes and multiple HMR operons in the chromosome (Fig. 2).

The presence of T6SS- and bacteriocin-related genes, both of which are involved in bacterial competition, in the Chr/Pla genes and fimbrial genes in both Chr/Pla and plasmid-specific genes may also be interesting, because inter-bacterial competition and fimbria-mediated adhesion can affect phenotypes that are important in niche colonization and specificity [41–43]. Some of the genes involved in bacterial competition may also be encoded by ICEs or other integrative elements, but a systematic and detailed analysis of these elements is required to clarify this issue.

### Pairwise Mash distance-based clustering of plasmids

**Fig. 5.**
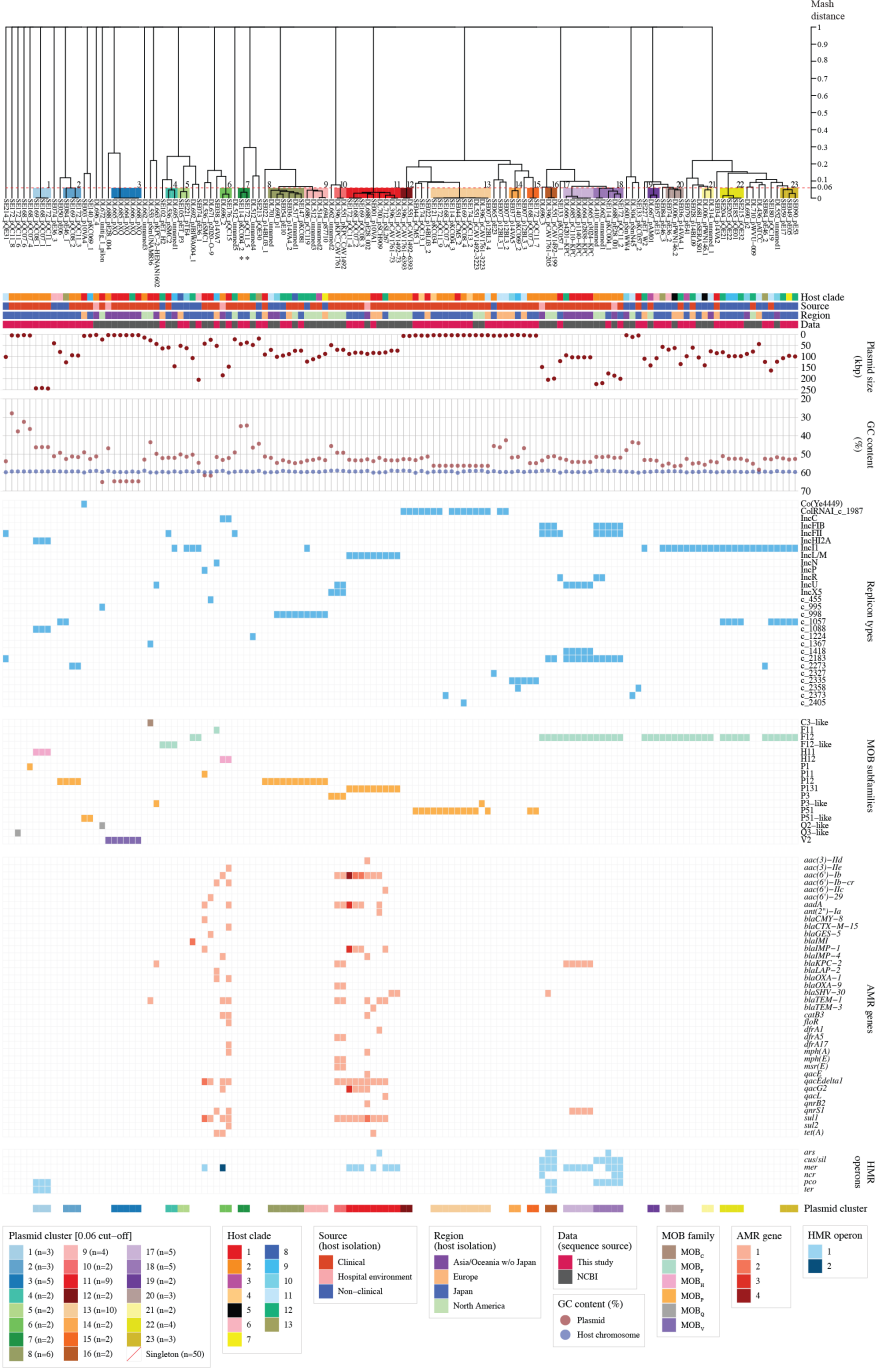
Pairwise Mash distance-based clustering of SMC plasmids. The 132 plasmids analysed in this study were separated into 23 clusters and 50 singletons with a cut-off distance of 0.06. Two linear plasmids are indicated by asterisks. Clades, isolation sources, regions of host strain isolation and sequence sources are shown along with the sizes and GC contents of plasmids, host chromosome GC content, replicon types and MOB subfamilies of plasmids, and carriage of AMR genes and HMR operons by each plasmid.

**Fig. 6.**
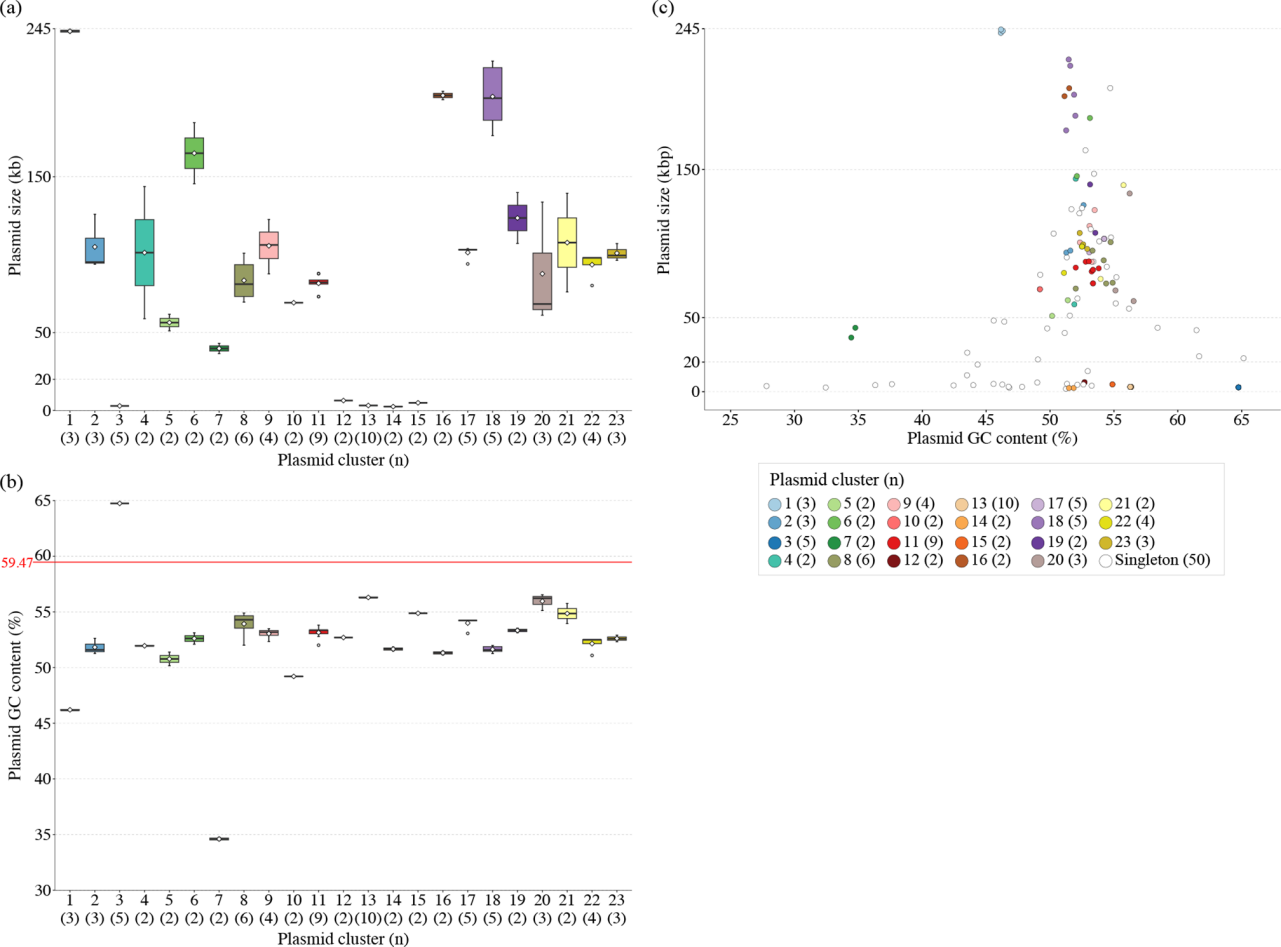
Sizes and GC contents of the 23 plasmid clusters and 50 singletons. The ranges of plasmid size (**a**) and GC content (**b**) in each plasmid cluster are shown. Open diamonds in the boxplot indicate mean values. In (**b**), average GC content of SMC chromosomes is also indicated by a red line. In (**c**), the relationship between the GC content and size of plasmids is shown. No significant correlation was observed in this comparison.

To analyse the similarity of the 132 plasmids, we performed a clustering analysis of these plasmids based on their pairwise Mash distances. With a threshold of 0.06 [3], the 132 plasmids were divided into 23 clusters that contained 2–10 members, and 50 singletons (Fig. 5). The lengths and GC contents of plasmids belonging to the same cluster were principally similar although some variations were observed in a few cases (Clusters 4, 20 and 21) (Figs 5 and 6). While the MOB types in the same cluster were concordant, the replicon types showed variations in eight clusters, but most variations observed were due to the presence or absence of a single replicon (Fig. 5 and Table S3). Of the 23 clusters, 11 consisted of plasmids from strains isolated in the same country: six in Japan, three in USA and two in China (Fig. S5a and Table S3). Of the remaining 12 clusters, 11 consisted of plasmids from strains isolated in two or three different continents and Cluster 4 comprised one plasmid from Japan and one from China (Fig. S5a and Table S3), indicating that the 11 plasmid clusters are globally distributed and not limited to a specific geographical region. Regarding the sources of host strains, clustered plasmids often shared the same source, clinical (including hospital environments) or non-clinical; 13 were exclusively found in clinical strains, and four were found only in non-clinical strains (Fig. 5; see Table S3 for details), suggesting the possible preference of plasmid distribution based on the environmental niches preferred by host strains, as previously observed with *Escherichia coli* plasmids [40]. Consistent with this, the carriage of AMR genes and HMR operons was also highly biased (Fig. 2), and these genes and operons were present in seven clusters and seven singletons, all of which were found in clinical/hospital environment strains except for one singleton (Fig. 5 and Table S3). Among these seven clusters, the plasmids in Clusters 6, 10 and 11 contained six or more different AMR genes and HMR operons (up to 14 AMR genes in one Cluster 6 plasmid) (Figs 5 and S6, and Table S3). Of note, the plasmids in Clusters 1, 16 and 18 carried two or more different HMR operons (up to four operons) but they carried no AMR genes except for one Cluster 16 plasmid that carried a single AMR gene (Figs 5 and S6, and Table S3). Thus, these plasmids can be regarded as specialized for HMR rather than AMR.

### Sizes and GC contents of plasmids

While 22 plasmids belonging to five clusters (Clusters 3, 12, 13, 14 and 15) and 17 singletons were smaller than 6.3 kb in size, most of the other plasmids (79/94) were larger than 50 kb (Figs 5 and 6a, and Table S3). In particular, Clusters 1, 6, 16 and 18 comprised very large plasmids (>176 kb, except for a 145.5 kb plasmid in Cluster 6). One singleton plasmid was also 204.9 kb. Thus, the carriage of these plasmids has a considerable impact on the total genome size of each strain, as suggested by our previous study [6]. As expected, the sizes of potentially self-transmissible plasmids were much larger (109 287 bp on average) than non-self-transmissible plasmids (16 076 bp on average). More importantly, most plasmid genomes showed notably lower GC contents than their host chromosomes (58.9–60.1 %; see Fig. S2), mostly ranging between 50 and 56 %, with extreme cases of the plasmids in Clusters 1 and 7, which showed approximately 46.2–46.3 and 34.4–34.8 % GC contents, respectively (Figs 5 and 6b, and Table S3). The GC contents of 11 singleton plasmids were also lower than 46 %, with one showing a 27.8 % GC content (Fig. 5). In addition, Cluster 3 plasmids showed much higher GC contents (64.8 %) than their host chromosomes (Figs 5 and 6b). The size and GC content of the 132 plasmids were not correlated (Fig. 6c), although smaller plasmids showed a wider range of GC contents than larger plasmids, as shown in an analysis of plasmids from a wide range of bacterial species [44]. These results suggest that the acquisitions of most plasmids (and/or their gene contents) by the strains analysed in this study were recent or relatively recent genetic events.

### Within-SMC transmission and potential host ranges of plasmids

**Fig. 7.**
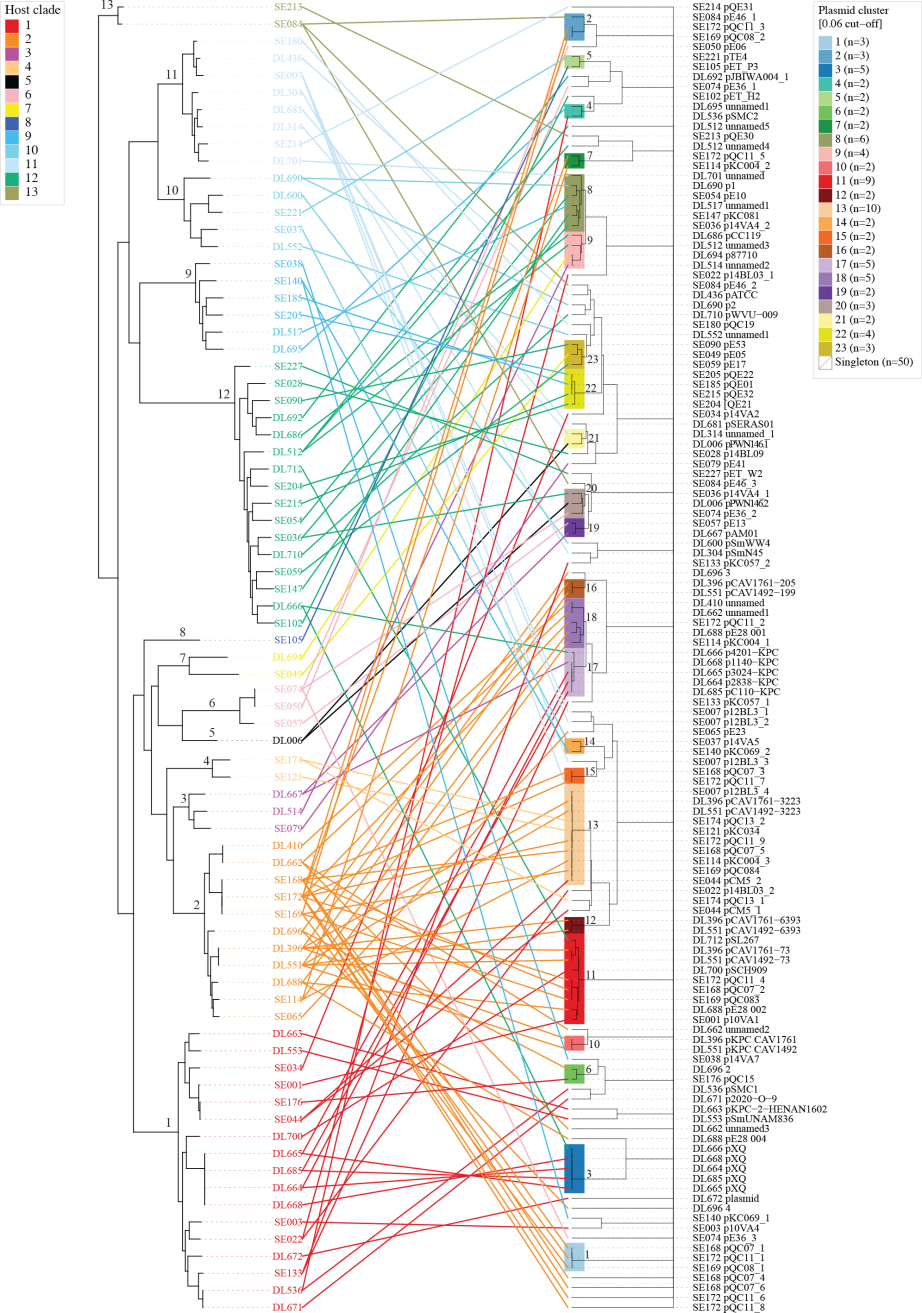
The correlation of plasmid clustering with host strain phylogeny. A core gene-based ML phylogenetic tree of the 77 plasmid-containing strains is presented along with the dendrogram showing the Mash distance-based clustering of the 132 plasmids found in the 77 strains. Plasmids and their hosts are linked by lines coloured according to host strain clades. The plasmid dendrogram was rotated to best mirror the host phylogenetic tree using the cophylo command from the phytools package in R v4.2.3. Host clades and plasmid clusters are indicated in the ML tree and the dendrogram, respectively.

Of the 23 plasmid clusters, 16 comprised plasmids distributed in two or more different host clades (Fig. 7, see also network diagrams of each plasmid cluster shown in Fig. S5b). Among these multiclade clusters, Clusters 8 and 13 consisted of plasmids from four clades (Fig. 7). This finding suggests the interclade transmission of the 16 plasmid clusters.

**Fig. 8.**
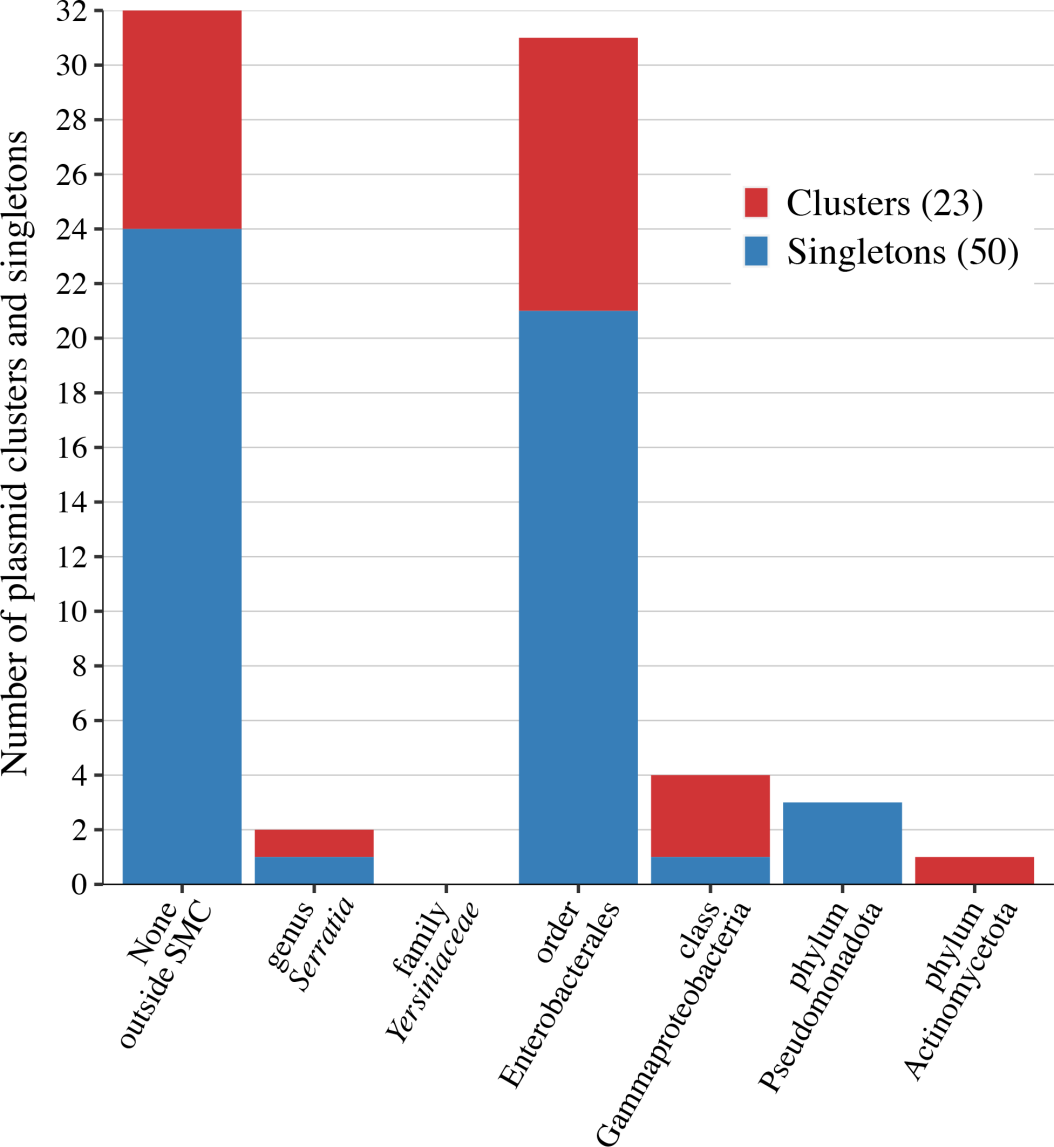
Potential host ranges of the 23 plasmid clusters and 50 singleton plasmids. Potential host ranges were predicted based on the detection of plasmids that were highly homologous (<0.06 Mash distance) to each plasmid cluster and singleton in the NCBI database (see Tables 3 and S5 for more details).

To analyse the potential host ranges of the SMC plasmids in a wider taxonomic scope, we first searched the NCBI database by BLASTn for plasmids similar to each of the 132 plasmids. Then, among the plasmid hits in this initial screening, we selected highly homologous plasmids with the same Mash distance threshold (0.06) as used for the clustering of SMC plasmids. This analysis identified 1955 plasmids outside the SMC (listed in Table S5), which should share very recent common ancestors with SMC plasmids, indicative of their potential transmission abilities. As summarized in Fig. 8 and Table 3, eight clusters and 24 singletons had no highly homologous plasmids in the database, and one cluster and one singleton had such plasmids only within the genus *Serratia *. However, for the plasmids in the other clusters and singletons, highly homologous plasmids were found in different species belonging to the order *Enterobacterales *, class *Gammaproteobacteria* or phylum *Pseudomonadota *. Moreover, a plasmid highly homologous to Cluster 15 was found in *Corynebacterium jeikeium *, a Gram-positive, rod-shaped bacterium belonging to the phylum *Actinomycetota* (Table 3). The species has been described as a skin colonizer of hospitalized patients but presents similar risk factors for infection as those for the SMC and was also isolated from hospital environments, similar to SMC strains [45]. This finding indicates that many SMC plasmids have a notably wide range of potential transmission abilities.

**Table 3.**
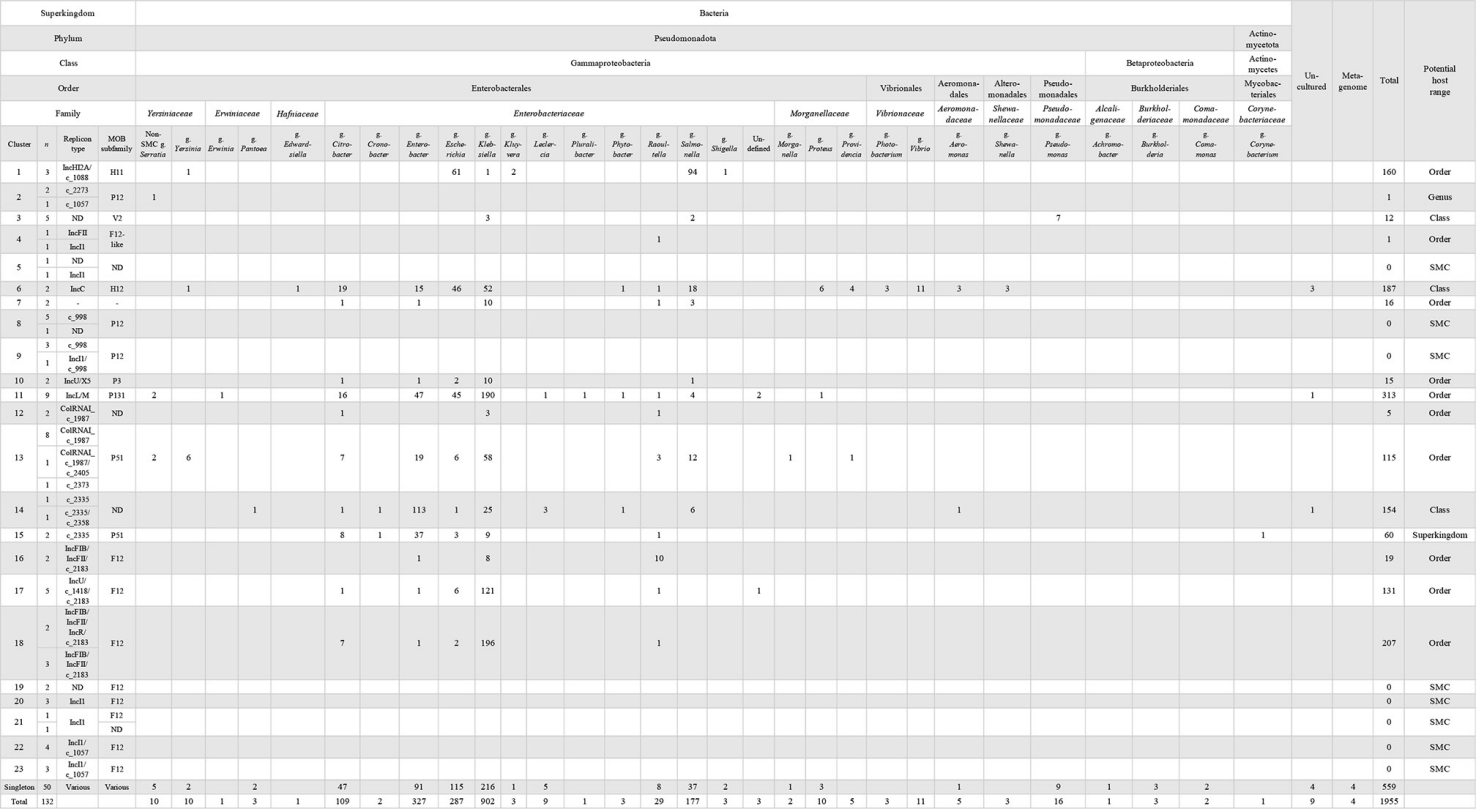
Potential host ranges of SMC plasmids

Potential host ranges were determined by the taxon level in which plasmids highly homologous to SMC plasmids were identified. c_XXXX; rep_cluster_ in MOB-typer, g.; genus.

Compared to the host ranges observed in this study, potential host ranges predicted by MOB-Typer based on MOB types were wider than our observation for 15 plasmid clusters and 31 singletons and the same as our observation for seven clusters and eight singletons (Table S6). However, it should be noted that the observed host ranges of seven singletons were wider than the MOB-Typer predictions and that one plasmid cluster (Cluster 3) and four singletons were unpredictable by MOB-Typer.

Importantly, many of the highly homologous plasmids identified in this study were found within the family *Enterobacteriaceae* (72.5 % of the 1955 plasmids; see Table S5 for details). Among these *Enterobacteriaceae* species, the most predominant were *Klebsiella* species (46.1 %), most of which were *K. pneumoniae* (38.8 %), followed by *Enterobacter* species (16.7 %; mostly *Enterobacter cloacae* and *Enterobacter hormaechei *) and *Escherichia coli* (14.6 %). Although the current database has a considerable species and genus bias, this finding suggests that these genera and species, particularly *K. pneumoniae *, may be the major sources for plasmid exchanges with the SMC, which may underlie the recent increase in MDR strains in the SMC and these *Enterobacteriaceae* species [5, 14, 46].

In a recent article describing the results of genomic analysis of the genus *Serratia* [10], Williams *et al*. also analysed *Serratia* plasmids including those from *S. marcescens* strains and reported that many of the *S. marcescens* plasmids have a predicted host range that goes beyond the taxonomic rank of family, including two clusters of small ColRNAI plasmids predicted to cross multiple phyla. These findings are consistent with the results obtained in this study.

## Conclusion

By analysing 142 closed genomes of SMC strains including 67 closed genomes obtained in this study, we identified 132 plasmids in 77 strains, which were separated into 23 clusters and 50 singletons based on their pairwise Mash distances. A systematic analysis of these plasmids revealed multiple important findings, including: (i) the identification of the genes specifically found in plasmids and those in chromosomes or plasmids, the latter of which included notable numbers of AMR and HMR genes that can move between chromosomes and plasmids; (ii) the notable difference in GC contents between most plasmids and their host chromosomes, which suggests their recent or relatively recent appearance in the SMC; (iii) the intra-SMC transmission of plasmids; and (iv) the broad potential host ranges of SMC plasmids beyond the genus, family, order, class or even phylum level. Moreover, members of the family *Enterobacteriaceae,* particularly *K. pneumoniae *, were suggested as the main sources for plasmid exchanges with the SMC. These results highlight the power of closed genome-based analysis in studies of plasmids and provide important insights into the nature of plasmids distributed in the SMC. The 67 closed genomes obtained in this study will also be important genomic resources for future studies of the SMC and other species in the genus *Serratia *, particularly for accurate analyses of their MGEs.

## Supplementary Data

Supplementary material 1

Supplementary material 2
