# Supplementary material for: Systematic analysis of plasmids of the Serratia marcescens complex using 142 closed genomes
Source: Microb Genom. 2023 Nov 15;9(11):001135. doi: 10.1099/mgen.0.001135 (PMC10711300; doi:10.1099/mgen.0.001135)
Supplement: Supplementary material 1 [file mgen-9-1135-s001.pdf]

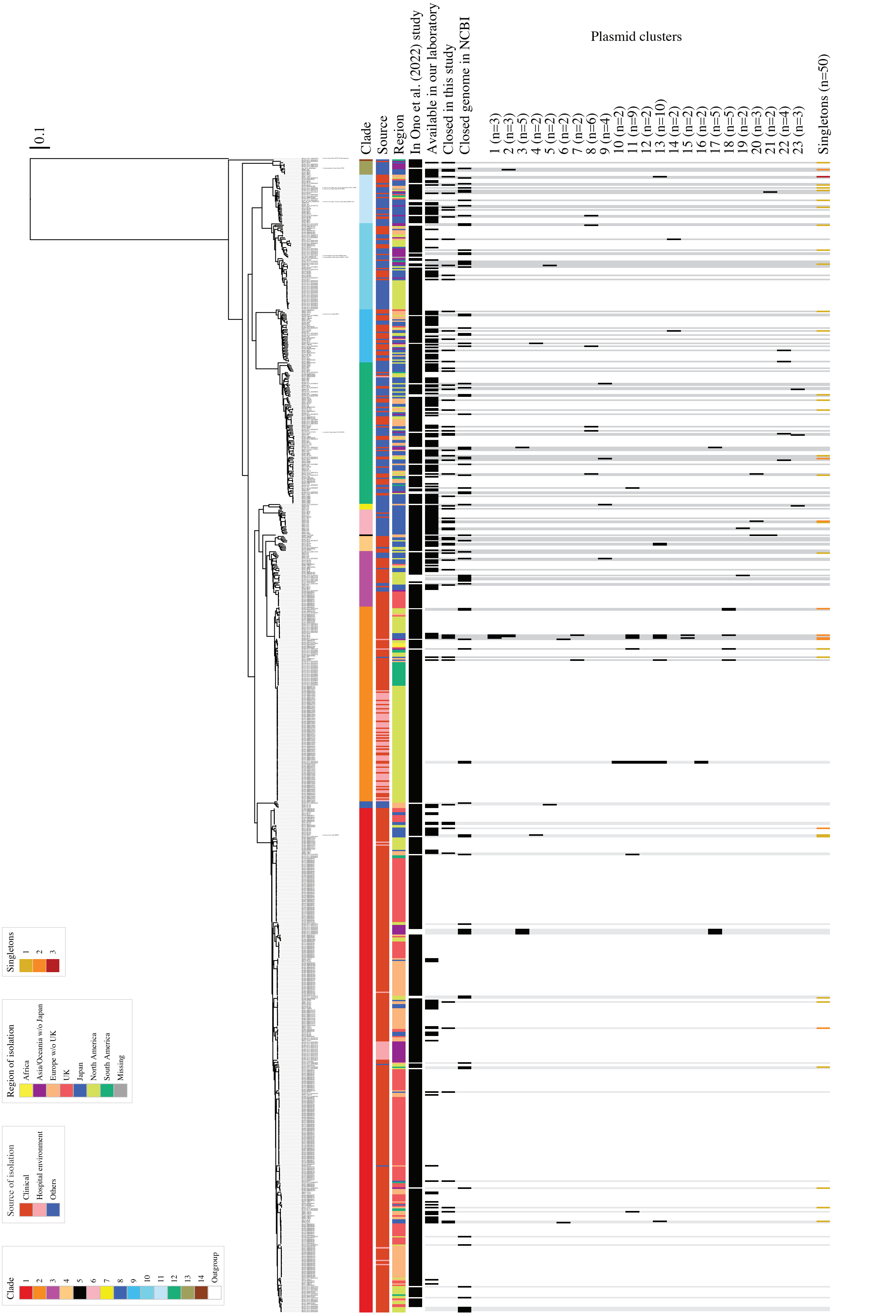

Fig. S1. Phylogenetic positions of the 142 genomes in the SMC. The core gene-based ML tree of the 775 SMC genomes that we previously analysed (Ono et al., 2022; Ref. 6 in the main text), 52 closed genomes and three draft genomes of type strains of three SMC species newly obtained from the NCBI database were constructed with a S. ficaria strain as an outgroup. The type strains and two SMC strains (Db11 and SM39) are indicated by their names. The clade, isolation source, isolation region are indicated for each strain. Strains analysed in the Ono et al. (2022) study, strains available in our laboratory, genomes closed in this study, and closed genomes obtained from NCBI are also indicated. The 142 closed genomes analysed in this study were highlighted by gray backgrounds and the distribution of plasmid clusters and singletons in these genomes were also indicated.

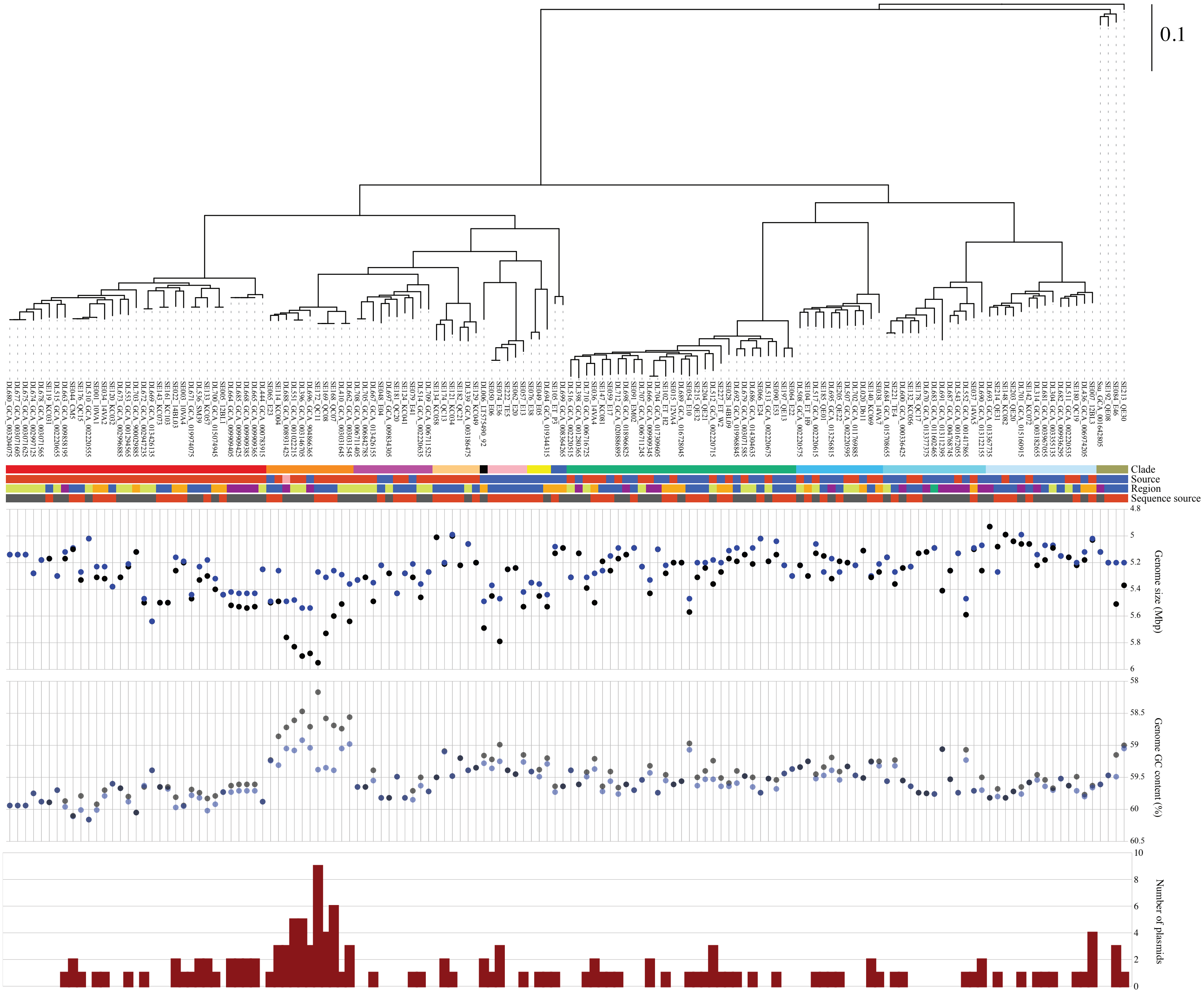

Clade: 1, 2, 3, 4, 5, 6, 7, 8, 9, 10, 11, 12, 13

Source of isolation: Clinical, Hospital environment, Others

Region of isolation: Asia/Oceania w/o Japan, Europe, Japan, North America, South America

Sequence source: This study, NCBI

Genomic parametes: Total genome size (Mbp), Chromosome size (Mbp), Total genome GC content (%), Chromosome GC content (%)

Genome size (Mbp)	Average	Range
Total genome	5.29	4.93 - 5.95
Chromosome	5.23	4.93 - 5.64

GC content (%)	Average	Range
Total genome	59.49	58.17 - 60.16
Chromosome	59.58	58.92 - 60.16

Fig. S2. Phylogenetic relationships of the 142 closed genomes analysed in this study. An ML tree was constructed based on the 535,875 SNPs identified in 3,189 core genes. The clade, isolation source, isolation region, genome sequence source (closed in this study and obtained from NCBI) are indicated for each genome. The sizes and GC content of the total genome and chromosome of each genome and the numbers of plasmids found in each genome are also shown.

(a)

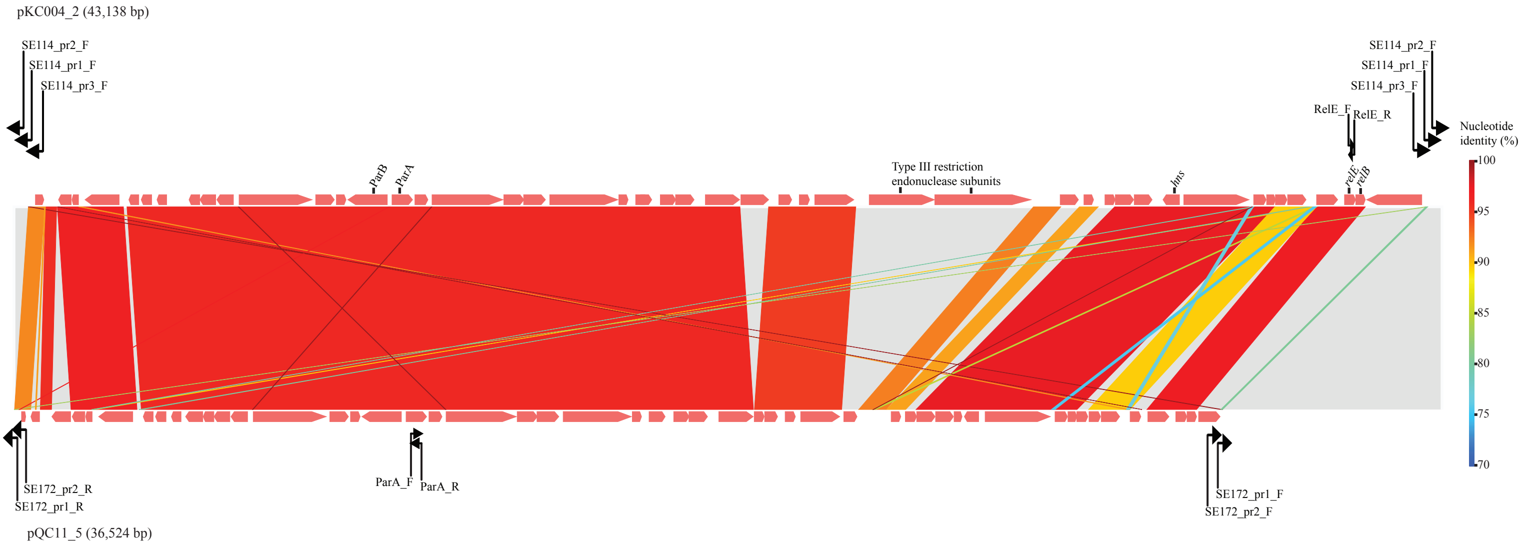

(b)

Agarose gel lane	Primers ID	Primers sequences (5' -> 3')	Expected size (bp)
A	SE114_pr1_F	CATGCACTTACATTCCTCTGAAC	
	SE114_pr1_R	TCGCCAGTTCCAGTATTTCC	
B	SE114_p2r_F	ATCCGGGTTTTACTCATACCA	
	SE114_pr2_R	TTCCTGATACCACATCGAATG	
C	SE114_pr3_F	ACACTTAATGAGCTGTCATCTGC	
	SE114_pr3_R	GCCAGCTTCTTGATCTGTTC	
D	RelE_F (positive control)	ATAGTAGGAACTGGCGGTTG	175
	RelE_R (positive control)	GAGCTGGTTTTTCTCTGCTG	
E	SE172_pr1_F	CTCAGGCTTCTCAGGTTCATTG	
	SE172_pr1_R	TCCCCCTGATAACATCGTACTATC	
F	SE172_pr2_F	GGGATGATTGGGAAAGGCATATAC	
	SE172_pr2_R	CAGGATAACTACTGGTTCAGAGTC	
G	ParA_F (positive control)	TGCGGAGAAAAACGGTAAAC	318
	ParA_R (positive control)	TTACGGATGTAGGGCTGTTC	

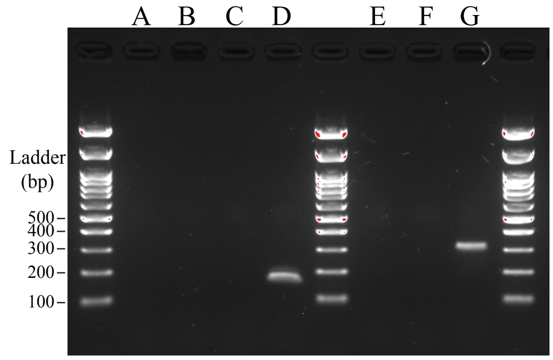

(c)

PFGE gel lane	Description	Circular plasmids (kb)	Linear plasmids (kb)
L8	PFGE standards 8 kb ladder		
L5	PFGE standards 5 kb ladder		
λ	Lambda PFG ladder		
Db	Strain Db11	-	-
E6	Strain E6	78.9	-
E36	Strain E36	204.9, 68.3 and 39.6	-
KC	Strain KC004	176.4 and 3.2	43.1
QC	Strain QC11	244.4, 200.4, 95.2, 87.9, 5.1, 4.9, 3.8 and 3.2	36.5

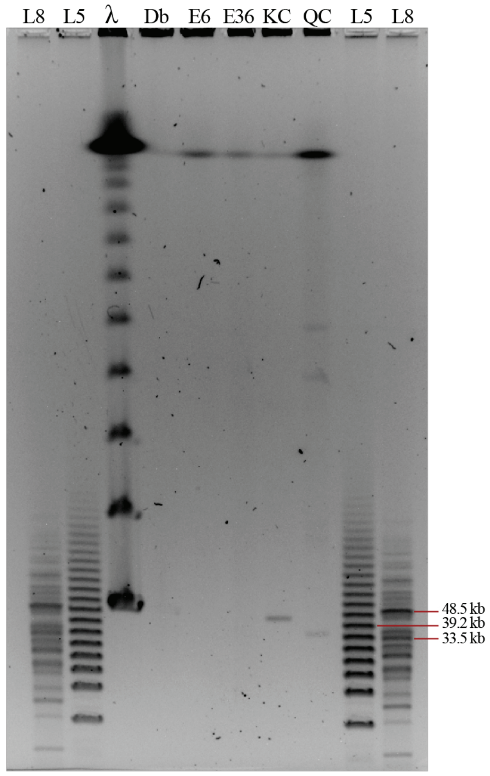

Fig. S3. Linear plasmids found in two SMC strains. (a) Genetic maps of the two linear plasmids and their nucleotide sequence identity are shown. These plasmids constituted Cluster 7. Genes indicated were previously described in similar linear plasmids from *Enterobacteriaceae* [37]. Positions and directions of the primers designed for PCR analysis shown in Panel (b) are also indicated. (b) Sequences of the PCR primers and the results of PCR analysis using these primers are shown. While the primer pair designed at the middle of the plasmid sequence yielded a band of the expected size in each strain (positive controls), the primer pairs targeting the ends of each plasmid sequence yielded no amplicons. (c) The result of PFGE analysis of undigested genomic DNA of the two strains carrying these linear plasmids is shown. A band with an expected size was detected in each strain, confirming the linearity of the two plasmids.

(a)

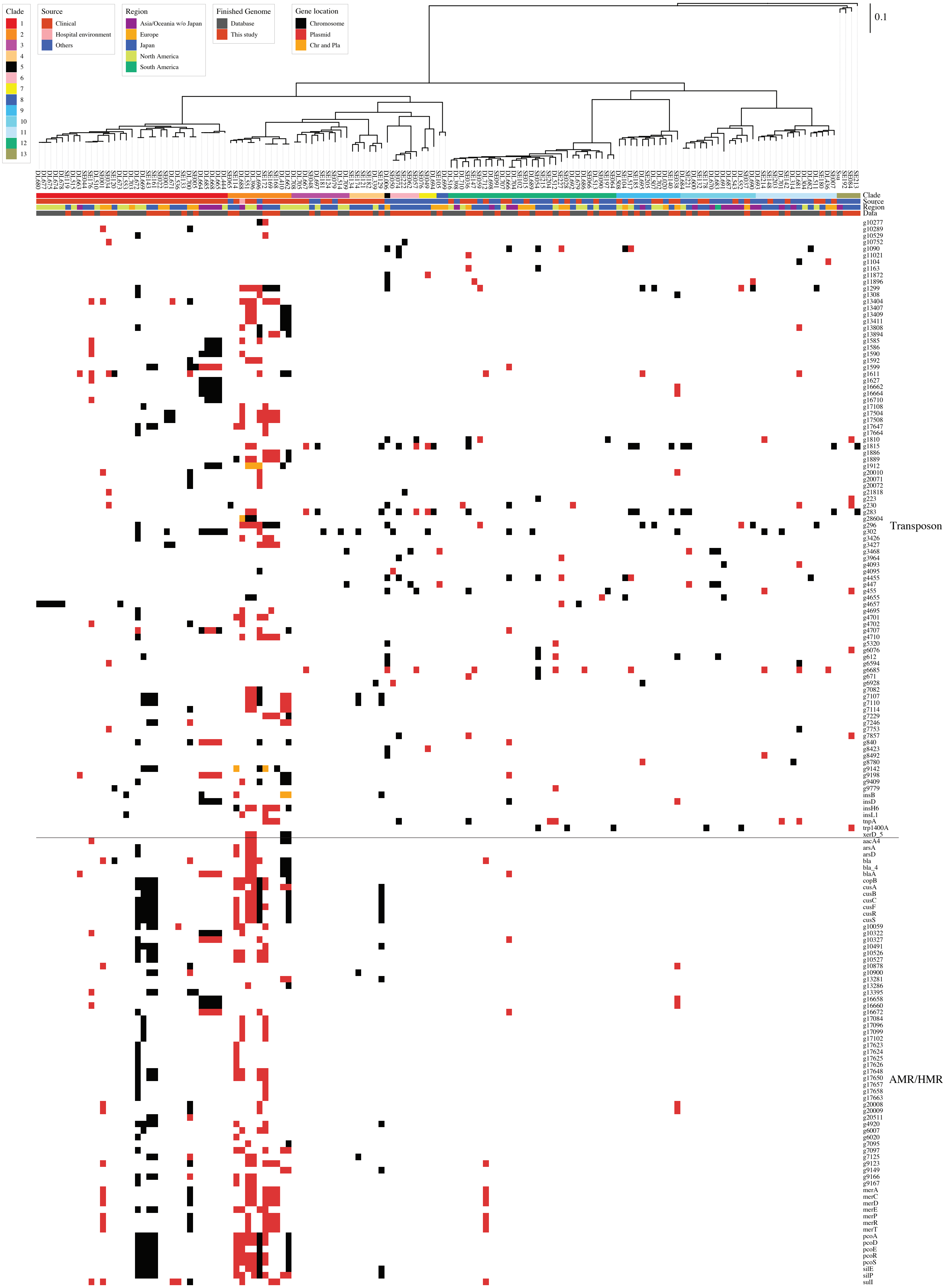

Clade
Source
Clinical
Hospital environment
Others
Region
Asia/Oceania w/o Japan
Europe
Japan
North America
South America
Finished Genome
Database
This study
Gene location
Chromosome
Plasmid
Chr and Pla
0.1
Clade
Source
Region
Data
Transposon
AMR/HMR

(b)

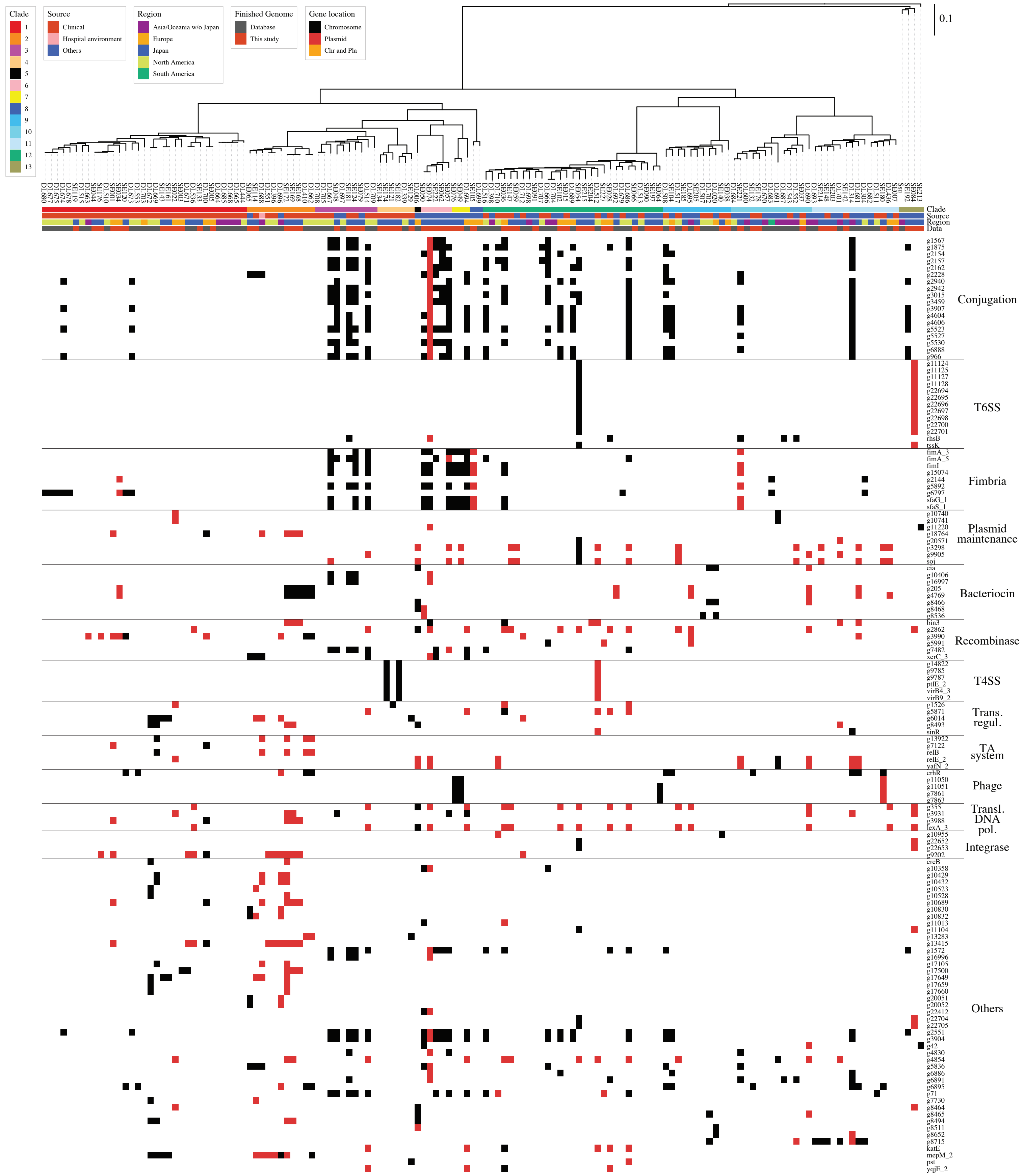

Fig. S4. Distribution of the 299 function-predicted Chr/Pla genes among the 142 closed SMC genomes. Genomic locations of each gene (chromosome or plasmid) are also indicated. Genes related to Transposon and AMR/HMR are shown in panel (a) and others are in panel (b). Trans. Regul.; Transcriptional Regulator, Transl. DNA pol.; Translesion DNA polymerase. See Table S4 for predicted functions of 299 genes.

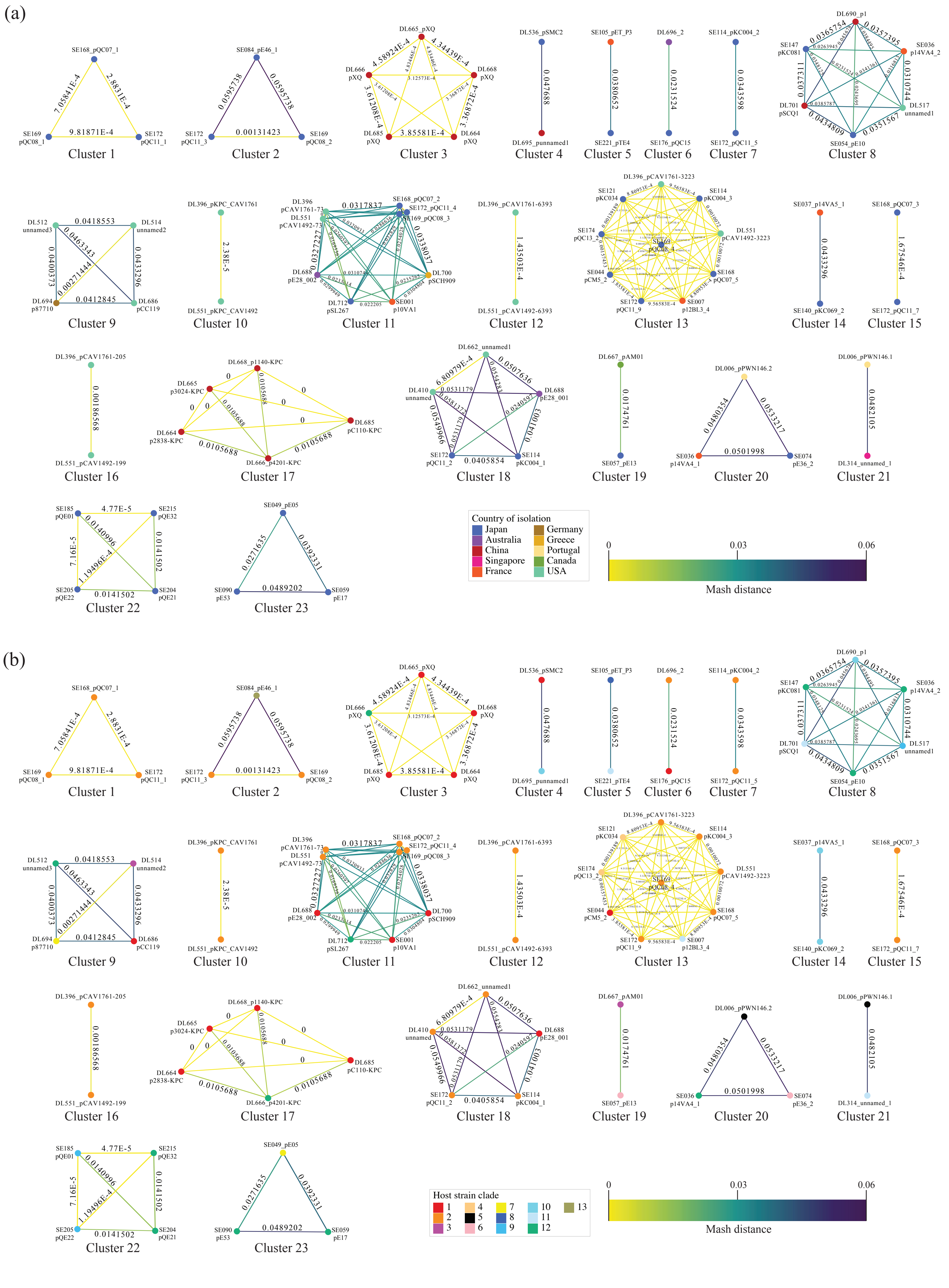

Fig. S5. Network diagrams of the 23 plasmid clusters prepared and visualized with Cytoscape v.3.10.0 [36] using Prefuse Force Directed layout weighted by pairwise Mash distances with default parameters. Geographic (country) information and clades of host strains are shown in (a) and (b), respectively.

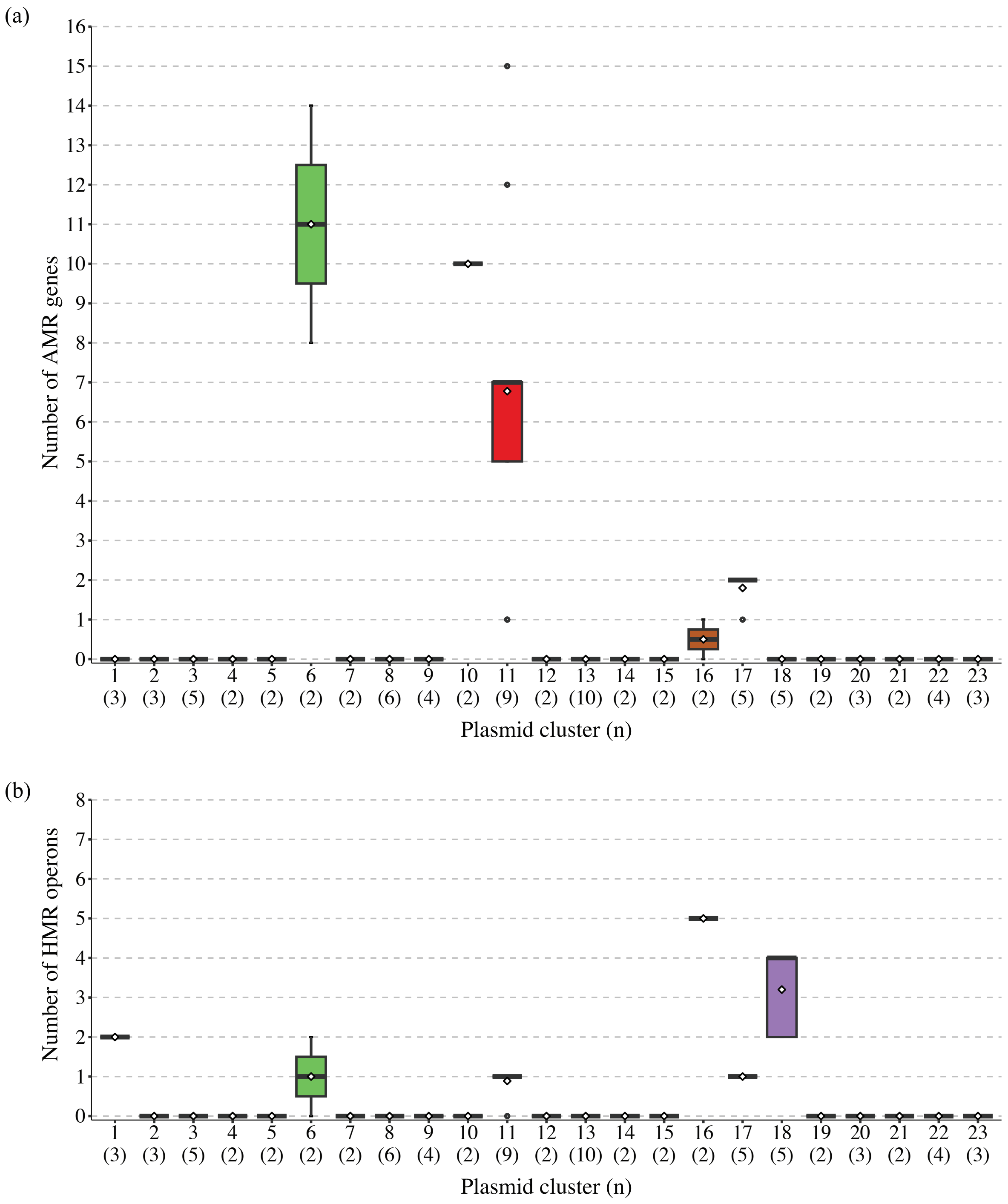

Fig. S6. The numbers of AMR genes (a) and HMR operons (b) encoded by each plasmid cluster.
